# An Integrated Work-Oriented Care Model Using Key Elements of the International Classification of Functioning and the Capability Approach

**DOI:** 10.5334/ijic.10270

**Published:** 2026-06-26

**Authors:** Desiree J. S. Dona, Suzanne E. J. Kaal, Bart P. A. Thoonen, Patrick Jeurissen, Frederieke G. Schaafsma

**Affiliations:** 1 Department of Primary and Community Care, Radboud university medical center, Geert Grooteplein 21, 6525 EZ Nijmegen, The Netherlands; 2 Department of Medical Oncology, Radboud university medical center, Geert Grooteplein-zuid 10, 6500 HB Nijmegen, The Netherlands; 3 Department of IQ Health, Radboud university medical center, Geert Grooteplein Zuid 10, 6525 GA Nijmegen, The Netherlands; 4 Department of Public and Occupational Health, Amsterdam UMC, Amsterdam Public Health research institute, The Netherlands

**Keywords:** biopsychosocial approach, Capability Approach (CA), International Classification of Functioning (ICF), work-oriented care, patient outcomes

## Abstract

Addressing the impact of chronic conditions on work participation requires an integrated care model that embeds work-related goals from diagnosis onward. The Work-oriented Care Model (WoCM) adopts a biopsychosocial, patient-centered approach and incorporates key elements of the Capability Approach (CA) and the International Classification of Functioning (ICF), aligning functional performance with individual goals and contextual factors. A case study illustrates the need for continuous work-oriented assessment involving healthcare and occupational professionals. Implementation challenges underscore the importance of structural integration and improved coordination across healthcare and occupational systems.

## Context and Aim

Chronic conditions often affect work capacity yet work participation as a treatment goal is frequently overlooked. This paper presents a Work-oriented Care Model (WoCM) that integrates work into clinical treatment and emphasizes collaboration with clinical occupational physicians (COPs) and nurses [1,2]. The model aims to bridge gaps between healthcare and occupational health by aligning patient goals with (clinical) decision-making and promoting early, coordinated action.

## Brief Description of Topic at Hand: The Work-Oriented Care Model

Developed and implemented at a Dutch university medical center, WoCM positions work as a meaningful and key component of treatment, rather than a peripheral concern [1,2]. Grounded in integrated care principles, WoCM ensures that work-related goals are embedded in treatment plans from diagnosis onward.

COPs are formal members of multidisciplinary treatment teams and contribute expertise in work participation during multidisciplinary team meetings (MDTs), which enable early identification and management of work-related challenges. COPs advise clinicians on incorporating work-related considerations into treatment strategies, facilitate timely referrals, and foster awareness of barriers to societal participation. This approach promotes shared responsibility and continuous interprofessional learning, positioning work as a recovery goal and as a therapeutic instrument supporting meaningful societal engagement.

WoCM’s effectiveness relies on structural integration of work-related expertise, proactive identification of work challenges, and recognition of work and socioeconomic security as integral aspects of health and well-being [3,4,5]. The WoCM combines patients’ individual goals and preferences regarding work with a biopsychosocial approach. It combines three inputs: 1) Patients’ input draws on the Capability Approach (CA), which focuses on what individuals can achieve under standardized conditions [6]; 2) Medical input encompasses diagnosis, treatment, and clinical outcomes; and 3) The biopsychosocial dimension based on the International Classification of Functioning (ICF), which broadens the perspective by considering dynamic interactions between health conditions, personal factors, and environmental influences [7,8]. The CA highlights capacities and opportunities, while ICF clarifies actual performance in everyday contexts. Together, these frameworks guide individualized Work-oriented Intervention Plans (WoIPs) [1].

## Analytic Approach to Work and Care

The ICF offers a holistic, multidimensional model to describe functioning and health-related conditions [7,8]. It divides functioning and participation into three perspectives: body, person, and environment. Using ICF, professionals organize information into two parts: 1) a patients’ functioning and ability to participate considering medical condition, body function or anatomical structure, and 2) contextual factors, split into environmental and personal factors. These factors act as barriers or enablers. Environmental factors are external elements like accessibility or social attitudes that affect functioning. Personal factors are individual traits such as motivation or coping style that influence how persons manage their condition. ICF distinguishes between resources (capacity in standardized settings) and performance (actual functioning in real-life contexts).

The CA emphasizes aligning individuals’ goals and capabilities with contextual factors [6]. Capabilities represent feasible opportunities shaped by personal abilities or capacities in conjunction with the environment and specific life circumstances, making CA highly relevant for integrated patient-centered care by considering the individual’s environment, such as the workplace, and thus offering a more realistic assessment of what patients can achieve within real-life settings. Its core concept — conversion factors — explains how resources become effective capabilities, distinguished by Robeyns (2017) and Van der Klink (2016) into organizational (e.g. company policies and societal legislation), work-related (e.g. work conditions), and personal factors (e.g. individual background, skills and motivation) [9,10]. Combining ICF and CA supports realistic assessments of patients’ possibilities in daily life and work.

This integrated basis strengthens the WoCM by ensuring systematic and continuous information gathering for the development of tailored WoIPs that integrate medical, personal, and contextual dimensions [1,2]. Joint application of ICF and CA allows WoCM to address dynamic interactions between health, environment, and preferences, promoting sustainable work participation. The following case study illustrates how this integrated approach functions in practice.

## Practical Application of the WoCM

A 51-year-old garden maintenance worker with a migration background, seeks treatment in the Netherlands after recurrent head and neck cancer. He values his job highly, but his primary concern is survival. No communication occurs between his employer and the occupational physician.

Following major surgery and re-irradiation, he experiences lasting impairments: impaired eyelid closure, restricted shoulder and arm function and fragile irradiated skin. Three months post-diagnosis, a nurse specialist — who performed an early brief work anamnesis — identifies work-related risks and refers him to the COP for a work-oriented assessment (Table 1). Using ICF and CA, the COP develops a WoIP. Whereas care initially focused solely on survival, the focus now shifts to rehabilitation and realistic work resumption. The WoIP includes targeted recommendations such as avoiding UV exposure, dusty environments and skin trauma. His job requires permanent adjustments that exclude outdoor and pruning tasks.

**Table 1:** Work-oriented assessment for patient with recurrent head and neck cancer.


ICF DOMAIN	HINDERING/PROMOTING FACTORS FOR PARTICIPATION

Health Condition (disorder or disease)	Recurrent head and neck cancer.

Body Structure and Functions	Permanent functional limitations due to surgery and re-irradiation: Nerve paralysis (eyelid closure, mouth skew). Tinnitus. Hearing loss. Restricted function shoulder girdle, neck and arm. Fragile skin in irradiated area. Dry mucous membranes in head and neck region.

Personal Factors	Moderately fluent in the Dutch language. Limited knowledge of Dutch social security legislation. Coping: grateful, optimistic, downplaying symptoms, socially desirable behavior. Work-related views: wants to keep his job but doubts feasibility, currently focused on health recovery, limited insight into potential return-to-work barriers.

Environmental Factors (work)	Elements of heavy physical work. Frequent overhand pruning. Dust exposure (risk due to impaired eyelid closure and damaged mucosa). Risk of traumatic injury to irradiated skin. Ultraviolet load on skin from outdoor work. Employer expects permanent disability pension.

Environmental Factors (private)	Wife runs a busy own business. Small social network.


An occupational therapist visits the workplace and discusses adaptations with the employer. After six months, his functional recovery makes work resumption realistic. The WoIP emphasizes rehabilitation while exploring feasible work options. Early, proactive adjustments guided by the WoCM support rehabilitation and sustainable return to work, preventing a potential permanent disability status [11,12,13], see Table 1.

## Discussion and Reflection

The case illustrates most clearly how the WoCM adds value: by integrating work considerations early and continuously, the treatment trajectory remains aligned with the patient’s evolving goals and functional possibilities. While frameworks such as ICF and CA provide essential conceptual structure, the strength of WoCM lies in how these frameworks inform practical, work-oriented treatment planning.

The case demonstrates that early attention to work prevents unnecessary long-term disability and maintain the therapeutic role of work in recovery. By identifying work-related risks — such as UV exposure, dust, and physical strain — during active medical treatment, the COP was able to influence both rehabilitation goals and workplace adaptations. This illustrates how work-oriented planning can and should occur while medical treatment is still ongoing, rather than after recovery is assumed.

The combination of ICF and CA guided the creation of a dynamic WoIP that aligned rehabilitation with sustainable work participation. ICF provided a structured overview of functional limitations and contextual barriers, while CA added insight into what was realistically achievable given personal motivation, workplace conditions, and available resources. This dual perspective helped the team shift from survival-oriented care to a pathway that maintained the therapeutic value of work and strengthened autonomy [14].

The case also shows how work preferences and goals evolve throughout a chronic disease trajectory. Patients move through phases, from diagnosis and uncertainty to adaptation and renewed engagement. WoCM supports these transitions by ensuring continuous reassessment rather than a ‘wait-and-see’ approach. Nurses, due to their close and ongoing contact, are well positioned to detect changes in health, goals, and environmental factors. Nurses facilitate this process by promoting health, functioning and quality of life, ensuring patients retain control over care [15]. Their long-term involvement strengthens the conversion of resources into practical opportunities.

Their collaboration with the COP ensures that work considerations remain an active part of the treatment conversation [2,16,17,18].

Successful implementation of WoCM requires structural conditions: the integration of COPs into multidisciplinary teams, systematic interprofessional learning, and sufficient resources for nurses to manage work-health complexity. At the same time, broader systemic barriers — such as policy constraints, economic insecurity, and persistent assumptions about work ability and chronic illness — continue to challenge sustainable participation [19,20]. By explicitly embedding work within healthcare, WoCM offers a practical strategy to counteract these barriers.

Overall, the case underscores that work-oriented treatment planning is not an add-on but a vital component of patient-centered care. It broadens the clinical focus from treating disease to supporting meaningful participation in life, during and after medical treatment.

## Conclusion and Recommendations

The WoCM offers a comprehensive framework for embedding work participation as a treatment goal within clinical care for patients with chronic conditions. By combining ICF and CA, healthcare professionals, can assess functional performance, individual goals, and contextual influences in an integrated manner, enabling realistic and motivating WoIPs incorporating personal resources, environmental factors, and autonomy.

The case study highlights how early and continuous attention to work-related goals prevents unnecessary long-term disability. As patient priorities change over time, the WoIP must be regularly updated to reflect new capabilities, insights, and workplace possibilities. The continuous involvement of COPs and nurses ensures that these shifts are recognized early and translated into actionable steps. For effective implementation of WoCM, healthcare systems should: 1) Integrate COPs structurally within treatment teams; 2) Promote interprofessional collaboration and learning to embed work-related thinking into routine care; 3) Equip nurses with expertise and resources to monitor work-health interactions and detect changes early; 4) Update WoIPs based on evolving patient needs and capacities; 5) Improve coordination among healthcare, work, and social systems to reduce fragmentation; 6) Address systemic barriers such as policy gaps, economic constraints, and stigma around chronic illness.

By framing work as a therapeutic and meaningful component of health, WoCM supports recovery, autonomy, and long-term societal participation. It shifts healthcare toward a model where work is not postponed until after treatment but actively integrated throughout the care trajectory [21].
